# Fossil Genera in Elateridae (Insecta, Coleoptera): A Triassic Origin and Jurassic Diversification

**DOI:** 10.3390/insects11060394

**Published:** 2020-06-26

**Authors:** Robin Kundrata, Gabriela Packova, Johana Hoffmannova

**Affiliations:** Department of Zoology, Faculty of Science, Palacky University, 17. listopadu 50, 77146 Olomouc, Czech Republic; gabriela.packova01@upol.cz (G.P.); johana.hoffmannova01@upol.cz (J.H.)

**Keywords:** classification, Cenozoic, click-beetles, Elateroidea, evolution, Mesozoic, palaeodiversity, systematics

## Abstract

Insect fossils bear important information about the evolutionary history of the group. The fossil record of Elateridae, a large cosmopolitan beetle family, has been greatly understudied and the available data are often replete with ambiguity and uncertainty. The research of Elateridae evolution cannot be done without solid genus-group name concepts. In this study we provide an updated comprehensive summary of the fossil genera in Elateridae, including their systematic placement and information on the type species, gender, number of species, age range, and relevant bibliography. We list seven valid fossil genera in Agrypninae, one in Cardiophorinae, two in Dendrometrinae, five in Elaterinae, two in Negastriinae, one in Omalisinae, one in Pityobiinae, and 36 in Protagrypninae. Additional 19 genera are tentatively classified as Elateridae *incertae sedis*, and their placements are discussed. Further, we move genera *Babuskaya* Martins-Neto & Gallego, 2009, *Cardiosyne* Martins-Neto & Gallego, 2006, *Fengningia* Hong, 1984 and *Gemelina* Martins-Neto & Gallego, 2006 from Elateridae to Coleoptera *incertae sedis*. We also discuss the genera previously placed in Elateridae, which are currently not included in the family. The data on the fossil generic diversity suggest that Elateridae originated in the Triassic and rapidly diversified and became comparatively abundant through the Jurassic. We call for further research on the fossil Elateridae from various deposits in order to increase our knowledge on the origin, evolution, and palaeodiversity of the group.

## 1. Introduction

Elateridae, or click-beetles, is a large beetle family containing about 10,000 described species worldwide [1]. This group includes several ecologically and economically important lineages, with some of them being among the most serious agricultural pests [2]. The current composition of Elateridae considerably differs from the historical concepts of the group, which usually included only hard-bodied lineages with a well-developed clicking mechanism, consisting of a prosternal process fitting into a mesoventral cavity [3,4,5,6]. With the rise of molecular phylogenetic analyses, several soft-bodied groups, which were previously considered separate families, i.e., Drilidae, Omalisidae, and Plastoceridae, were merged with Elateridae [7,8]. However, the internal phylogenetic relationships, as well as the suprageneric classification of the group, remain open to further study [9,10,11].

Elateridae have a long evolutionary history which dates back to the early Mesozoic [12,13,14,15,16,17]. Numerous taxa were reported mainly from the Jurassic deposits of China, Kazakhstan, and Kyrgyzstan [13,17,18,19,20,21,22], but the rich elaterid fossil record is known also from various Cretaceous sedimentary deposits and amber [16,23,24,25,26,27,28]. The Cenozoic Elateridae fossils are known mainly from the Eocene deposits of the United States and Europe [29,30,31], and from the Baltic amber [32]. Altogether, the fossil record of Elateridae includes approximately 300 species classified in more than 100 genera [28,33]. Regarding the fossil genera in Elateridae, Scudder [34] published the World catalogue of the fossil insects which included also taxa assigned to this family. Handlirsch [35] listed in Elateridae many doubtful taxa which can belong to various other beetle families. Hyslop [36] was the first who attempt to cover all genus-group names in Elateridae. He also catalogued the fossil genera, including those previously listed by Handlirsch [35]. Dolin [13] revised the fossil record of Elateridae from the Jurassic of Karatau (Karabastau Formation of Kazakhstan). Spahr [37] published the catalogue of beetles from amber and copal. Carpenter [38] compiled the list of fossil genera in Coleoptera, including Elateridae, and placed many dubious taxa into Coleoptera *incertae sedis*. Alekseev [39] provided the checklist of beetles described from the Baltic amber. The online open checklist of fossil beetles by Kirejtshuk and Ponomarenko [33] includes also Elateridae but requires some updates, including changes in classification. Most recently, the first part of the World catalogue of the genus-group names in Elateridae contained only extant taxa [40].

Fossils represent an important data source for understanding morphological character evolution, elucidating the relationships among lineages, and dating the phylogenetic divergence events. However, it is crucial to understand the taxonomic and phylogenetic position of fossil taxa in order to correctly interpret the evolutionary history of the group. Consequently, misinterpretations and misidentifications of fossils often result in incorrect conclusions [15,16]. Such research cannot be carried out without the solid genus-group name concepts which are consistent with regulations from the International Commission on Zoological Nomenclature [41]. In this study, we provide a summary of all fossil genus-group names including type species and their designations, misspellings, correct gender, and their systematic position according to the most recent publications. This study could serve as a starting point for future research on the click-beetle palaeodiversity and evolution.

## 2. Materials and Methods

We provide information on all fossil genus-group names in Elateridae, including their current systematic status. The family classification follows that of Kundrata et al. [9], with subsequent changes made by Kusy et al. [8] and Kundrata et al. [10]. Generally, we follow the style used in the first part of the World catalogue of the genus-group names in Elateridae [40]. The names of the family-, genus- and (type) species-group taxa are given with the name of the author, and the year and page of publication. The page given is the page where the taxon name and description are printed. The year and page given for the incorrect subsequent spellings are the first year and page in which they are used. Incorrect subsequent spellings not in prevailing usage are unavailable according to Art. 33.3 of the Code [41]. We provide the type species for each genus-group name, including information on its designation. Misspellings are followed by colon “:”. Taxa marked with an asterisk (*) contain also recent species. Only fossil genera are listed, except for the recent *Athous* Eschscholtz, 1829, *Ampedus* Dejean, 1833 and *Limonius* Eschscholtz, 1829, each of which contains one fossil subgenus. The age of fossils was taken from the Paleobiology Database (https://paleobiodb.org/). Divisions of geological time and their boundaries follow the ICS International Chronostratigraphic Chart v. 2020/01 (http://www.stratigraphy.org/) [42]. The overview of the fossil genera and subgenera in Elateridae is summarized in Table 1.

## 3. Results

Family Elateridae Leach, 1815*

Elaterides Leach, 1815: 85 [43]. Type genus: *Elater* Linnaeus, 1758. For more information, including synonyms, see Bouchard et al. [44] and Kundrata et al. [40].

### 3.1. Overview of the Fossil Genera and Subgenera in Elateridae

#### 3.1.1. Subfamily Agrypninae Candèze, 1857*

Agrypnides Candèze, 1857: 17 [45]. Type genus: *Agrypnus* Eschscholtz, 1829. For more information, including synonyms, see Kundrata et al. [40].

- Tribe Agrypnini Candèze, 1857*

Agrypnides Candèze, 1857: 17 [45]. Type genus: *Agrypnus* Eschscholtz, 1829. For more information, including synonyms, see Kundrata et al. [40].

Genus *Ageratus* Dolin, 1980

*Ageratus* Dolin, 1980: 72 [13]. Gender: masculine. Type species: *Ageratus ponomarenkoi* Dolin, 1980: 73 [13]; by original designation. Jurassic of Kazakhstan (166.1–157.3 Ma). Two species.

Literature. Dolin (1980: 72) [13], Carpenter (1992: 304) [38], Korneev & Cate (2005: 9) [46], Alekseev (2011: 424) [26].

Genus *Compsoderus* Dolin, 1980

*Compsoderus* Dolin, 1980: 71 [13]. Gender: masculine. Type species: *Compsoderus priscus* Dolin, 1980: 72 [13]; by original designation. Jurassic of Kazakhstan (166.1–157.3 Ma). Monotypic.

*Compsoferus*: Carpenter, 1992: 304 [38]; unavailable name, incorrect subsequent spelling not in prevailing usage [41].

Literature. Dolin (1980: 71) [13], Carpenter (1992: 304) [38], Korneev & Cate (2005: 10) [46].

Genus *Litholacon* Dolin, 1980

*Litholacon* Dolin, 1980: 67 [13]. Gender: masculine. Type species: *Litholacon derumpens* Dolin, 1980: 68 [13]; by original designation. Jurassic of Kazakhstan (166.1–157.3 Ma). Seven species.

Literature. Dolin (1980: 67) [13], Carpenter (1992: 305) [38], Korneev & Cate (2005: 10) [46], Alekseev (2011: 424) [26].

Genus *Macropunctum* Tröster, 1991

*Macropunctum* Tröster, 1991: 100 [31]. Gender: neuter. Type species: *Macropunctum messelense* Tröster, 1991: 106 [31]; by original designation. Eocene of Germany (48.6–40.4 Ma), Eocene of the United Kingdom (38.0–33.9 Ma). 13 species.

Literature. Tröster (1991: 100) [31], Tröster (1992: 111) [47], Tröster (1993: 49) [48], Tröster (1994a: 58) [49], Tröster (1994b: 145) [50], Tröster (1999: 12) [51], Wappler (2003: 86) [52], Kirejtshuk et al. (2019: 453) [53].

Genus *Plagioraphes* Iablokoff-Khnzorian, 1961

*Plagioraphes* Iablokoff-Khnzorian, 1961: 84 [32]. Gender: masculine. Type species: *Plagioraphes fasciatus* Iablokoff-Khnzorian, 1961: 85 [32]; by original designation. Eocene Baltic amber (38.0–33.9 Ma). Monotypic.

Literature. Iablokoff-Khnzorian (1961: 84) [32], Larsson (1978: 153) [54], Spahr (1981: 49) [37], Carpenter (1992: 305) [38], Alekseev (2013: 7) [39], Alekseev (2017: 409) [55].

Remark. This genus was originally placed in Agrypninae [32]. Alekseev [39] listed *Plagioraphes* under Pityobiinae but without any justification or clarification. Here, we follow the original placement by Iablokoff-Khnzorian [32].

- Tribe Cryptocardiini Dolin, 1980

Cryptocardiini Dolin, 1980: 74 [13]. Type genus: *Cryptocardius* Dolin, 1980.

Genus *Cryptocardius* Dolin, 1980

*Cryptocardius* Dolin, 1980: 74 [13]. Gender: masculine. Type species: *Cryptocardius mirabilis* Dolin, 1980: 75 [13]; by original designation. Jurassic of Kazakhstan (166.1–157.3 Ma). Monotypic.

Literature. Dolin (1980: 74) [13], Carpenter (1992: 304) [38], Korneev & Cate (2005: 10) [46], Chang et al., (2007: 1248) [56], Alekseev (2011: 423) [26].

- Tribe Pyrophorini Candèze, 1863*

Pyrophorites Candèze, 1863: 3 [57]. Type genus: *Pyrophorus* Billberg, 1820.

Genus *Eopyrophorus* Haupt, 1950

*Eopyrophorus* Haupt, 1950: 101 [30]. Gender: masculine. Type species: *Eopyrophorus mixtus* Haupt, 1950: 107 [30]; by monotypy. Eocene of Germany (47.8–41.3 Ma). Monotypic.

Literature. Haupt (1950: 101) [30], Haupt (1956: 48) [58], Kinzelbach & Lutz (1985: 600) [59], Carpenter (1992: 304) [38].

#### 3.1.2. Subfamily Cardiophorinae Candèze, 1859*

Cardiophorites Candèze, 1859: 4 [60]. Type genus: *Cardiophorus* Eschscholtz, 1829. For more information, including synonyms, see Bouchard et al. [44].

Genus *Mionelater* Becker, 1963

*Mionelater* Becker, 1963: 125 [61]. Gender: masculine. Type species: *Mionelater planatus* Becker, 1963: 126 [61]; by original designation. Miocene of Mexico (Chiapas amber) (23.0–16.0 Ma). Monotypic.

Literature. Becker (1963: 125) [61], Spahr (1981: 49) [37], Carpenter (1992: 305) [38], Solórzano Kraemer (2007: 120) [62], Douglas (2017: 50) [63].

Remark. Douglas [63] suggested that based on the shapes of head, posterior pronotal angles and mesocoxal cavities, this genus might belong to a different subfamily.

#### 3.1.3. Subfamily Dendrometrinae Gistel, 1848*

Dendrometridae Gistel, 1848: 5 [64]. Type genus: *Dendrometrus* Gistel, 1848. For more information, including synonyms, see Bouchard et al. [44].

- Tribe Dendrometrini Reitter, 1905*

Dendrometridae Gistel, 1848: 5 [64]. Type genus: *Dendrometrus* Gistel, 1848. For more information, including synonyms, see Bouchard et al. [44].

Genus *Athous* Eschscholtz, 1829*

*Athous* Eschscholtz, 1829: 33 [65]. Type species: *Elater vittatus* Fabricius, 1792: 224 [66]; by subsequent designation (Westwood 1838: 26) [67]. For more information, including synonyms, see Sánchez-Ruiz [68] and Cate [69].

Subgenus *Athousiomorphus* Iablokoff-Khnzorian, 1961

*Athousiomorphus* Iablokoff-Khnzorian, 1961: 92 [32]. Gender: masculine. Type species: *Athous* (*Athousiomorphus*) *olgae* Iablokoff-Khnzorian, 1961: 92 [32]; by original designation. Eocene Baltic amber (38.0–33.9 Ma). Monotypic.

*Athousimorphus*: Alekseev, 2013: 7 [39]; unavailable name, incorrect subsequent spelling not in prevailing usage [41].

Literature. Iablokoff-Khnzorian (1961: 92) [32], Larsson (1978: 153) [54], Spahr (1981: 46) [37], Alekseev (2013: 7) [39].

Genus *Limonius* Eschscholtz, 1829*

*Limonius* Eschscholtz, 1829: 33 [65]. Type species: *Elater minutus* Linnaeus, 1758: 406 [70]; by subsequent designation (Curtis 1838: 694) [71]. For more information, including synonyms, see Sánchez-Ruiz [68] and Cate [69].

Subgenus *Paralimonius* Iablokoff-Khnzorian, 1961

*Paralimonius* Iablokoff-Khnzorian, 1961: 91 [32]. Gender: masculine. Type species: *Limonius* (*Paralimonius*) *barovskyi* Iablokoff-Khnzorian, 1961: 91 [32]; by original designation. Eocene Baltic amber (38.0–33.9 Ma). Monotypic.

Literature. Iablokoff-Khnzorian (1961: 91) [32], Larsson (1978: 153) [54], Spahr (1981: 48) [37], Alekseev (2013: 7) [39].

- Tribe Dimini Candèze, 1863*

Dimites Candèze, 1863: 237 [57]. Type genus: *Dima* Charpentier, 1825.

Genus *Alaodima* Dolin, 1980

*Alaodima* Dolin, 1980: 75 [13]. Gender: feminine. Type species: *Alaodima grandis* Dolin, 1980: 76 [13]; by original designation. Jurassic of Kazakhstan (166.1–157.3 Ma). Monotypic.

Literature. Dolin (1980: 75) [13], Carpenter (1992: 304) [38], Korneev & Cate (2005: 9) [46], Kundrata et al., (2018: 68) [72].

- Tribe Hypnoidini Schwarz, 1906*

Hypnoidini Schwarz, 1906: 150 [3]. Type genus: *Hypnoidus* Dillwyn, 1829.

Genus *Cryptagriotes* Wickham, 1916

*Cryptagriotes* Wickham, 1916: 512 [29]. Gender: masculine. Type species: *Cryptagriotes minusculus* Wickham, 1916: 512 [29]; by original designation. Eocene of the United States (37.2–33.9 Ma). Monotypic.

Literature. Wickham (1916: 512) [29], Hyslop (1921: 637) [36], Carpenter (1992: 304) [38].

#### 3.1.4. Subfamily Elaterinae Leach, 1815*

Elaterides Leach, 1815: 85 [43]. Type genus: *Elater* Linnaeus, 1758. For more information, including synonyms, see Bouchard et al. [44].

- Tribe Ampedini Fleutiaux, 1947*

Ampedidae Gistel, 1848: 5 [64]. Type genus: *Ampedus* Dejean, 1833.

Genus *Ampedus* Dejean, 1833*

*Ampedus* Dejean, 1833: 92 [73]. Type species: *Elater sanguineus* Linnaeus, 1758: 405 [70]; by subsequent designation (Curtis 1838: 694) [71]. For more information, including synonyms, see Sánchez-Ruiz [68] and Cate [69].

Subgenus *Octamenogonoides* Iablokoff-Khnzorian, 1961

*Octamenogonoides* Iablokoff-Khnzorian, 1961: 88 [32]. Gender: masculine. Type species: *Elater* (*Octamenogonoides*) *gebleri* Iablokoff-Khnzorian, 1961: 88 [32]; by original designation. Eocene Baltic amber (38.0–33.9 Ma). Monotypic.

*Octamenagonoides*: Alekseev, 2013: 7 [39]; unavailable name, incorrect subsequent spelling not in prevailing usage [41].

Literature. Iablokoff-Khnzorian (1961: 88) [32], Spahr (1981: 48) [37], Alekseev (2013: 7) [39].

Remark. It was originally described as a subgenus of *Elater* Linnaeus, 1758 [32]. Alekseev [39] transferred *Octamenogonoides* to *Ampedus* Dejean, 1833.

- Tribe Elaterini Leach, 1815*

Elaterides Leach, 1815: 85 [43]. Type genus: *Elater* Linnaeus, 1758. For more information, including synonyms, see Bouchard et al. [44].

Genus *Diaraphes* Iablokoff-Khnzorian, 1961

*Diaraphes* Iablokoff-Khnzorian, 1961: 89 [32]. Gender: masculine. Type species: *Diaraphes kozhantshikovi* Iablokoff-Khnzorian, 1961: 89 [32]; by original designation. Eocene Baltic amber (38.0–33.9 Ma). Monotypic.

Literature. Iablokoff-Khnzorian (1961: 89) [32], Larsson (1978: 153) [54], Spahr (1981: 47) [37], Carpenter (1992: 304) [38], Alekseev (2013: 7) [39], Alekseev (2017: 409) [55].

Genus *Elatron* Iablokoff-Khnzorian, 1961

*Elatron* Iablokoff-Khnzorian, 1961: 90 [32]. Gender: neuter. Type species: *Elatron semenovi* Iablokoff-Khnzorian, 1961: 90 [32]; by original designation. Eocene Baltic amber (38.0–33.9 Ma). Monotypic.

Literature. Iablokoff-Khnzorian (1961: 90) [32], Larsson (1978: 153) [54], Spahr (1981: 48) [37], Carpenter (1992: 304) [38], Alekseev (2013: 7) [39], Alekseev (2017: 409) [55].

Genus *Holopleurus* Iablokoff-Khnzorian, 1961

*Holopleurus* Iablokoff-Khnzorian, 1961: 86 [32]. Gender: masculine. Type species: *Holopleurus succineus* Iablokoff-Khnzorian, 1961: 86 [32]; by original designation. Eocene Baltic amber (38.0–33.9 Ma). Monotypic.

*Holopeurus*: Carpenter, 1992: 304 [38]; unavailable name, incorrect subsequent spelling not in prevailing usage [41].

Literature. Iablokoff-Khnzorian (1961: 86) [32], Larsson (1978: 153) [54], Spahr (1981: 48) [37], Carpenter (1992: 304) [38], Alekseev (2013: 7) [39], Alekseev (2017: 409) [55].

Genus *Orthoraphes* Iablokoff-Khnzorian, 1961

*Orthoraphes* Iablokoff-Khnzorian, 1961: 86 [32]. Gender: masculine. Type species: *Orthoraphes reichardti* Iablokoff-Khnzorian, 1961: 87 [32]; by original designation. Eocene Baltic amber (38.0–33.9 Ma). Monotypic.

Literature. Iablokoff-Khnzorian (1961: 86) [32], Larsson (1978: 153) [54], Spahr (1981: 49) [37], Carpenter (1992: 305) [38], Alekseev (2013: 7) [39], Alekseev (2017: 409) [55].

- Elaterinae incertae sedis

Genus *Crioraphes* Iablokoff-Khnzorian, 1961

*Crioraphes* Iablokoff-Khnzorian, 1961: 93 [32]. Gender: masculine. Type species: *Crioraphes rohdendorfi* Iablokoff-Khnzorian, 1961: 94 [32]; by original designation. Eocene Baltic amber (38.0–33.9 Ma). Monotypic.

Literature. Iablokoff-Khnzorian (1961: 93) [32], Larsson (1978: 153) [54], Spahr (1981: 46) [37], Carpenter (1992: 304) [38], Douglas (2011: 17) [74], Alekseev (2013: 7) [39], Alekseev (2017: 409) [55], Douglas (2017: 4) [63].

Remark. This genus was originally placed in Cardiophorinae [32], however, Douglas [74] transferred it to Elaterinae *incertae sedis* based on the morphological phylogenetic analysis.

#### 3.1.5. Subfamily Negastriinae Nakane & Kishii, 1956*

Negastriinae Nakane & Kishii, 1956: 203 [75]. Type genus: *Negastrius* Thomson, 1859.

Genus *Ganestrius* Dolin, 1976

*Ganestrius* Dolin, 1976: 69 [20]. Gender: masculine. Type species: *Ganestrius stibicki* Dolin, 1976: 71 [20]; by original designation. Jurassic of Kazakhstan (166.1–157.3 Ma). Two species.

Literature. Dolin (1976: 69) [20], Dolin (1980: 77) [13], Carpenter (1992: 304) [38], Korneev & Cate (2005: 10) [46].

Genus *Protoquasimus* Dolin, 1976

*Protoquasimus* Dolin, 1976: 69 [20]. Gender: masculine. Type species: *Protoquasimus brevicollis* Dolin, 1976: 69 [20]; by original designation. Jurassic of Kazakhstan (166.1–157.3 Ma). Monotypic.

Literature. Dolin (1976: 69) [20], Dolin (1980: 76) [13], Carpenter (1992: 305) [38], Korneev & Cate (2005: 10) [46].

#### 3.1.6. Subfamily Omalisinae Lacordaire, 1857*

Homalisides Lacordaire, 1857: 303 [76]. Type genus: *Omalisus* Geoffroy, 1762.

Genus *Jantarokrama* Kirejtshuk & Kovalev, 2015

*Jantarokrama* Kirejtshuk & Kovalev, 2015: 1413 [77]. Gender: feminine. Type species: *Jantarokrama utilis* Kirejtshuk & Kovalev, 2015: 1414 [77]; by original designation. Eocene Baltic amber (38.0–33.9 Ma). Monotypic.

Literature. Kirejtshuk & Kovalev (2015: 1413) [77], Alekseev (2017: 410) [55].

#### 3.1.7. Subfamily Pityobiinae Hyslop, 1917*

Pityobiinae Hyslop, 1917: 249 [78]. Type genus: *Pityobius* LeConte, 1853.

Genus *Cretopityobius* Otto, 2019

*Cretopityobius* Otto, 2019: 4 [27]. Gender: masculine. Type species: *Cretopityobius pankowskiorum* Otto, 2019: 4 [27]; by original designation. Cretaceous of Myanmar (Burmese amber) (99.6–93.5 Ma). Monotypic.

Literature. Otto (2019: 4) [27].

#### 3.1.8. Subfamily Protagrypninae Dolin, 1973

Protagrypnini Dolin, 1973: 74 [18]. Type genus: *Protagrypnus* Dolin, 1973.

- Tribe Desmatini Dolin, 1975

Desmatini Dolin, 1975: 60 [19]. Type genus: *Desmatus* Dolin, 1975.

Genus *Anoixis* Chang, Kirejtshuk & Ren, 2010

*Anoixis* Chang, Kirejtshuk & Ren, 2010: 872 [25]. Gender: feminine. Type species: *Anoixis complanus* Chang, Kirejtshuk & Ren, 2010: 873 [25]; by original designation. Cretaceous of China (125.5–122.5 Ma). Monotypic.

Literature. Chang et al. (2010: 872) [25], Dong & Huang (2011: 1225) [79], Yu et al. (2019: 383) [80].

Genus *Apoclion* Chang, Kirejtshuk & Ren, 2010

*Apoclion* Chang, Kirejtshuk & Ren, 2010: 869 [25]. Gender: masculine. Type species: *Apoclion clavatus* Chang, Kirejtshuk & Ren, 2010: 870 [25]; by original designation. Cretaceous of China (125.5–122.5 Ma). Three species.

Literature. Chang et al. (2010: 869) [25], Dong & Huang (2011: 1225) [79], Yu et al. (2019: 383) [80].

Genus *Desmatinus* Chang, Kirejtshuk & Ren, 2010

*Desmatinus* Chang, Kirejtshuk & Ren, 2010: 868 [25]. Gender: masculine. Type species: *Desmatinus cognatus* Chang, Kirejtshuk & Ren, 2010: 869 [25]; by original designation. Cretaceous of China (125.5–122.5 Ma). Monotypic.

Literature. Chang et al. (2010: 868) [25], Dong & Huang (2011: 1225) [79], Yu et al. (2019: 383) [80].

Genus *Desmatus* Dolin, 1975

*Desmatus* Dolin, 1975: 60 [19]. Gender: masculine. Type species: *Desmatus lapidarius* Dolin, 1975: 61 [19]; by original designation. Jurassic of Kazakhstan (166.1–157.3 Ma). Four species.

Literature. Dolin (1975: 60) [19], Dolin (1980: 64) [13], Carpenter (1992: 304) [38], Korneev & Cate (2005: 10) [46], Chang et al. (2008: 60) [81], Chang et al. (2009: 8) [17], Chang et al. (2010: 869) [25].

Genus *Paradesmatus* Chang, Kirejtshuk & Ren in Chang et al., 2009

*Paradesmatus* Chang, Kirejtshuk & Ren in Chang et al., 2009: 8 [17]. Gender: masculine. Type species: *Paradesmatus baie* Chang, Kirejtshuk & Ren, 2009: 8 [17]; by original designation. Cretaceous of China (125.5–122.5 Ma), Jurassic of China (166.1–157.3 Ma). Three species.

Literature. Chang et al. (2009: 8) [17], Chang et al. (2010: 867) [25], Kirejtshuk et al. (2010: 791) [82], Dong & Huang (2011: 1225) [79], Yu et al. (2019: 382) [80].

Genus *Plesiorhaphes* Dolin, 1980

*Plesiorhaphes* Dolin, 1980: 65 [13]. Gender: masculine. Type species: *Plesiorhaphes scaber* Dolin, 1980: 66 [13]; by original designation. Jurassic of Kazakhstan (166.1–157.3 Ma). Monotypic.

*Plesioraphes*: Korneev & Cate, 2005: 10 [46]; unavailable name, incorrect subsequent spelling not in prevailing usage [41].

Literature. Dolin (1980: 65) [13], Carpenter (1992: 305) [38], Korneev & Cate (2005: 10) [46], Chang et al. (2008: 60) [81], Chang et al. (2010: 867) [25].

- Tribe Hypnomorphini Dolin, 1975

Hypnomorphini Dolin, 1975: 54 [19]. Type genus: *Hypnomorphus* Dolin, 1975.

Hipnomorphini: Ponomarenko et al., 2012: 482 [83]; unavailable name, incorrect subsequent spelling not in prevailing usage [41].

Genus *Abrotus* Dolin, 1980

*Abrotus* Dolin, 1980: 62 [13]. Gender: masculine. Type species: *Abrotus sepultus* Dolin, 1980: 63 [13]; by original designation. Jurassic of Kazakhstan (166.1–157.3 Ma). Two species.

Literature. Dolin (1980: 62) [13], Carpenter (1992: 304) [38], Korneev & Cate (2005: 9) [46], Chang et al. (2008: 60) [81], Chang et al. (2011: 36) [84].

Remark. The non-type species of this genus was considered a member of Cerophytidae by Chang et al. [84], and the authors also called for a re-examination of the type species to test the systematic position of *Abrotus*.

Genus *Adiagnostus* Dolin, 1980

*Adiagnostus* Dolin, 1980: 44 [13]. Gender: masculine. Type species: *Adiagnostus cardiophorinus* Dolin, 1980: 45 [13]; by original designation. Jurassic of Kazakhstan (166.1–157.3 Ma). Three species.

Literature. Dolin (1980: 44) [13], Carpenter (1992: 304) [38], Korneev & Cate (2005: 9) [46], Chang et al. (2008: 60) [81], Alekseev (2011: 424) [26].

Genus *Codemus* Dolin, 1980

*Codemus* Dolin, 1980: 35 [13]. Gender: masculine. Type species: *Codemus synaptoides* Dolin, 1980: 36 [13]; by original designation. Jurassic of Kazakhstan (166.1–157.3 Ma). 10 species.

Literature. Dolin (1980: 35) [13], Carpenter (1992: 304) [38], Korneev & Cate (2005: 10) [46], Chang et al. (2008: 60) [81], Alekseev (2011: 424) [26].

Genus *Elaterophanes* Handlirsch, 1906

*Elaterophanes* Handlirsch, 1906: 436 [35]. Gender: masculine. Type species: *Elater socius* Giebel, 1856: 91 [85] (synonym of *Elater vetustus* Brodie, 1845: 101 [12]); by subsequent designation (Hyslop 1921: 644 [36]). Later designations by Whalley [14] (*E. vetustus*) and Carpenter [38] (*E. socius*) are invalid. Triassic of the United Kingdom (208.5–201.3 Ma), Jurassic of the United Kingdom (196.5–189.6 Ma). Three species.

Literature. Handlirsch (1906: 436) [35], Cockerell (1915: 478) [86], Hyslop (1921: 644) [36], Dolin (1973: 73) [18], Dolin (1975: 51) [19], Whalley (1985: 165) [14], Carpenter (1992: 304) [38].

Genus *Graciolacon* Dolin, 1980

*Graciolacon* Dolin, 1980: 61 [13]. Gender: masculine. Type species: *Graciolacon aeternus* Dolin, 1980: 62 [13]; by original designation. Jurassic of Kazakhstan (166.1–157.3 Ma). Monotypic.

Literature. Dolin (1980: 61) [13], Carpenter (1992: 304) [38], Korneev & Cate (2005: 10) [46], Chang et al. (2008: 60) [81].

Genus *Hypnomorphoides* Dolin, 1980

*Hypnomorphoides* Dolin, 1980: 54 [13]. Gender: masculine. Type species: *Hypnomorphoides catachtonius* Dolin, 1980: 55 [13]; by original designation. Jurassic of Kazakhstan (166.1–157.3 Ma). Four species.

Literature. Dolin (1980: 54) [13], Carpenter (1992: 304) [38], Korneev & Cate (2005: 10) [46], Chang et al. (2008: 60) [81].

Genus *Hypnomorphus* Dolin, 1975

*Hypnomorphus* Dolin, 1975: 54 [19]; erroneously published as gen. nov. also by Dolin (1980: 26) [13]. Gender: masculine. Type species: *Hypnomorphus rohdendorfi* Dolin, 1975: 56 [19]; by original designation. Jurassic of Kazakhstan (166.1–157.3 Ma). 14 species.

*Hyponomorphus*: Chang et al., 2008: 60 [81]; unavailable name, incorrect subsequent spelling not in prevailing usage [41].

Literature. Dolin (1975: 54) [19], Dolin (1980: 26) [13], Carpenter (1992: 304) [38], Korneev & Cate (2005: 10) [46], Chang et al. (2008: 60) [81], Dong et al. (2011: 482) [87].

Genus *Idiomorphus* Dolin, 1980

*Idiomorphus* Dolin, 1980: 60 [13]. Gender: masculine. Type species: *Idiomorphus singularis* Dolin, 1980: 60 [13]; by original designation. Jurassic of Kazakhstan (166.1–157.3 Ma). Two species.

Literature. Dolin (1980: 60) [13], Carpenter (1992: 304) [38], Korneev & Cate (2005: 10) [46], Chang et al. (2008: 60) [81].

Genus *Lapidiconides* Dolin, 1980

*Lapidiconides* Dolin, 1980: 43 [13]. Gender: masculine. Type species: *Lapidiconides excellens* Dolin, 1980: 43 [13]; by original designation. Jurassic of Kazakhstan (166.1–157.3 Ma). Three species.

*Lapidioconides*: Korneev & Cate: 10 [46]; unavailable name, incorrect subsequent spelling not in prevailing usage [41].

Literature. Dolin (1980: 43) [13], Carpenter (1992: 304) [38], Korneev & Cate (2005: 10) [46], Chang et al. (2008: 60) [81].

Genus *Lapidostenus* Dolin, 1980

*Lapidostenus* Dolin, 1980: 30 [13]. Gender: masculine. Type species: *Lapidostenus infossus* Dolin, 1980: 31 [13]; by original designation. Jurassic of Kazakhstan (166.1–157.3 Ma). Five species.

Literature. Dolin (1980: 30) [13], Carpenter (1992: 304) [38], Korneev & Cate (2005: 10) [46], Chang et al. (2008: 60) [81].

Genus *Lithoptychus* Dolin, 1980

*Lithoptychus* Dolin, 1980: 57 [13]. Gender: masculine. Type species: *Lithoptychus handlirschi* Dolin, 1980: 57 [13]; by original designation. Jurassic of Kazakhstan (166.1–157.3 Ma). Four species.

Literature. Dolin (1980: 57) [13], Carpenter (1992: 305) [38], Korneev & Cate (2005: 10) [46], Chang et al. (2008: 60) [81].

Genus *Lithosomus* Dolin, 1980

*Lithosomus* Dolin, 1980: 46 [13]. Gender: masculine. Type species: *Lithosomus erosus* Dolin, 1980: 47 [13]; by original designation. Jurassic of Kazakhstan (166.1–157.3 Ma). Two species.

Literature. Dolin (1980: 46) [13], Carpenter (1992: 305) [38], Korneev & Cate (2005: 10) [46], Chang et al. (2008: 60) [81].

Genus *Necrocoelus* Dolin, 1980

*Necrocoelus* Dolin, 1980: 59 [13]. Gender: masculine. Type species: *Necrocoelus aselloides* Dolin, 1980: 59 [13]; by original designation. Jurassic of Kazakhstan (166.1–157.3 Ma). Monotypic.

*Necrocelus*: Carpenter, 1992: 305 [38]; unavailable name, incorrect subsequent spelling not in prevailing usage [41].

Literature. Dolin (1980: 59) [13], Carpenter (1992: 305) [38], Korneev & Cate (2005: 10) [46], Chang et al. (2008: 60) [81].

Genus *Negastrioides* Dolin, 1980

*Negastrioides* Dolin, 1980: 52 [13]. Gender: masculine. Type species: *Negastrioides tenuis* Dolin, 1980: 52 [13]; by original designation. Jurassic of Kazakhstan (166.1–157.3 Ma). Four species.

*Negastroides*: Korneev & Cate, 2005: 15 [46]; unavailable name, incorrect subsequent spelling not in prevailing usage [41].

Literature. Dolin (1980: 52) [13], Carpenter (1992: 305) [38], Korneev & Cate (2005: 10) [46], Chang et al. (2008: 60) [81].

Genus *Parahypnomorphus* Dolin, 1980

Parahypnomorphus Dolin, 1980: 33 [13]. Gender: masculine. Type species: *Parahypnomorphus jurassicus* Dolin, 1980: 33 [13]; by original designation. Jurassic of Kazakhstan (166.1–157.3 Ma). Three species.

Literature. Dolin (1980: 33) [13], Carpenter (1992: 305) [38], Korneev & Cate (2005: 10) [46], Chang et al. (2008: 60) [81].

Genus *Platyelater* Dolin, 1980

*Platyelater* Dolin, 1980: 40 [13]. Gender: masculine. Type species: *Platyelater reflexicollis* Dolin, 1980: 41 [13]; by original designation. Jurassic of Kazakhstan (166.1–157.3 Ma). Four species.

*Platyelata*: Carpenter, 1992: 305 [38]; unavailable name, incorrect subsequent spelling not in prevailing usage [41].

Literature. Dolin (1980: 40) [13], Carpenter (1992: 305) [38], Korneev & Cate (2005: 10) [46], Chang et al. (2008: 60) [81], Dong et al. (2011: 482) [87].

- Tribe Pollostelaterini Alekseev, 2011

Pollostelaterini Alekseev, 2011: 424 [26]. Type genus: *Pollostelater* Alekseev, 2011.

Pollostelasterini: Sohn et al., 2019: 9 [28]; unavailable name, incorrect subsequent spelling not in prevailing usage [41].

Genus *Pollostelater* Alekseev, 2011

*Pollostelater* Alekseev, 2011: 424 [26]. Gender: masculine. Type species: *Pollostelater baissensis* Alekseev, 2011: 424 [26]; by original designation. Cretaceous of Russian Federation (125.0–113.0 Ma). Monotypic.

Literature: Alekseev (2011: 424) [26].

- Tribe Protagrypnini Dolin, 1973

Protagrypnini Dolin, 1973: 74 [18]. Type genus: *Protagrypnus* Dolin, 1973.

Genus *Acheonus* Dolin, 1980

*Acheonus* Dolin, 1980: 20 [13]. Gender: masculine. Type species: *Acheonus abbreviatus* Dolin, 1980: 21 [13]; by original designation. Jurassic of Kazakhstan (166.1–157.3 Ma). Three species.

*Archeonus*: Carpenter, 1992: 304 [38]; unavailable name, incorrect subsequent spelling not in prevailing usage [41].

Literature. Dolin (1980: 20) [13], Carpenter (1992: 304) [38], Korneev & Cate (2005: 9) [46], Chang et al. (2008: 60) [81], Alekseev (2011: 424) [26], Dong et al. (2011: 482) [87].

Genus *Archaeolus* Lin, 1986

*Archaeolus* Lin, 1986: 78 [21]. Gender: masculine. Type species: *Archaeolus funestus* Lin, 1986: 78 [21]; by original designation. Jurassic of China (170.3–168.3 Ma). Monotypic.

Literature: Lin (1986: 78) [21], Zhang (1997: 71) [88], Dolin & Nel (2002: 345) [89], Dong & Huang (2009: 102) [22], Dong & Huang (2011: 1225) [79], Dong et al. (2011: 481) [87], Ponomarenko et al. (2012: 479) [83], Yu et al. (2019: 409) [80].

Genus *Clavelater* Dong & Huang, 2011

*Clavelater* Dong & Huang, 2011: 1225 [79]. Gender: masculine. Type species: *Clavelater ningchengensis* Dong & Huang, 2011: 1226 [79]; by monotypy. Jurassic of China (166.1–157.3 Ma). Monotypic.

Literature: Dong & Huang (2011: 1225) [79], Yu et al. (2019: 383) [80].

Genus *Koreagrypnus* Sohn & Nam in Sohn et al., 2019

*Koreagrypnus* Sohn & Nam in Sohn et al., 2019: 6 [28]. Gender: masculine. Type species: *Koreagrypnus jinju* Sohn & Nam in Sohn et al., 2019: 6 [28]; by original designation. Cretaceous of South Korea (113.0–100.5 Ma). Monotypic.

Literature. Sohn et al. (2019: 6) [28].

Genus *Lithocoelus* Dolin, 1975

*Lithocoelus* Dolin, 1975: 53 [19]. Gender: masculine. Type species: *Lithocoelus detrusus* Dolin, 1975: 53 [19]; by original designation. Jurassic of Kazakhstan (166.1–157.3 Ma). Two species.

Literature. Dolin (1975: 53) [19], Dolin (1980: 20) [13], Carpenter (1992: 305) [38], Korneev & Cate (2005: 10) [46], Chang et al. (2008: 60) [81].

Genus *Lithomerus* Dolin, 1980

*Lithomerus* Dolin, 1980: 23 [13]. Gender: masculine. Type species: *Lithomerus cockerelli* Dolin, 1980: 23 [13]; by original designation. Jurassic of Australia (182.7–174.1 Ma), Jurassic of Kazakhstan (166.1–157.3 Ma), Cretaceous of China (125.5–122.5 Ma). Six species.

Literature. Dolin (1980: 23) [13], Carpenter (1992: 305) [38], Dolin & Nel (2002: 341) [89], Korneev & Cate (2005: 10) [46], Chang et al. (2008: 55) [81], Dong & Huang (2009: 103) [22], Kirejtshuk et al. (2010: 792) [82], Martin (2010: 932) [90], Dong & Huang (2011: 1225) [79], Ponomarenko et al. (2012: 479) [83], Sohn et al. (2019: 3) [28], Yu et al. (2019: 382) [80].

Genus *Megalithomerus* Sohn & Nam in Sohn et al., 2019

*Megalithomerus* Sohn & Nam in Sohn et al., 2019: 3 [28]. Gender: masculine. Type species: *Megalithomerus magohalmii* Sohn & Nam in Sohn et al., 2019: 3 [28]; by original designation. Cretaceous of South Korea (113.0–100.5 Ma). Monotypic.

Literature. Sohn et al. (2019: 3) [28].

Genus *Micragrypnites* Dolin, 1973

*Micragrypnites* Dolin, 1973: 76 [18]. Gender: masculine. Type species: *Micragrypnites issykiensis* Dolin, 1973: 77 [18]; by original designation. Jurassic of Kyrgyzstan (201.3–190.8 Ma). Monotypic.

Literature. Dolin (1973: 76) [18], Dolin (1975: 51) [19], Carpenter (1992: 305) [38], Korneev & Cate (2005: 10) [46], Chang et al. (2008: 60) [81], Dong & Huang (2009: 103) [22], Dong et al. (2011: 482) [87].

Genus *Paragrypnites* Dolin, 1980

*Paragrypnites* Dolin, 1980: 22 [13]. Gender: masculine. Type species: *Paragrypnites jagemanni* Dolin, 1980: 22 [13]; by original designation. Jurassic of Kazakhstan (166.1–157.3 Ma). Monotypic.

*Paragrypnus*: Chang et al., 2008: 60 [81]; unavailable name, incorrect subsequent spelling not in prevailing usage [41].

Literature. Dolin (1980: 22) [13], Carpenter (1992: 305) [38], Korneev & Cate (2005: 10) [46], Chang et al. (2008: 60) [81], Ponomarenko et al. (2012: 479) [83].

Genus *Paraprotagrypnus* Chang, Zhao & Ren, 2009

*Paraprotagrypnus* Chang, Zhao & Ren, 2009: 1433 [91]. Gender: masculine. Type species: *Paraprotagrypnus superbus* Chang, Zhao & Ren, 2009: 1434 [91]; by original designation. Jurassic of China (166.1–157.3 Ma). Monotypic.

Literature. Chang et al. (2009: 1433) [91], Kirejtshuk et al. (2010: 791) [82], Dong & Huang (2011: 1225) [79], Sohn et al. (2019: 3) [28], Yu et al. (2019: 382) [80].

Genus *Protagrypnus* Dolin, 1973

*Protagrypnus* Dolin, 1973: 75 [18]. Gender: masculine. Type species: *Protagrypnus exoletus* Dolin, 1973: 75 [18]; by original designation. Jurassic of Kyrgyzstan (201.3–190.8 Ma), Jurassic of China (166.1–157.3 Ma). Two species.

*Protagrypnites*: Chang et al., 2008: 60 [81]; unavailable name, incorrect subsequent spelling not in prevailing usage [41].

Literature. Dolin (1973: 75) [18], Dolin (1975: 51) [19], Carpenter (1992: 305) [38], Korneev & Cate (2005: 10) [46], Chang et al. (2008: 60) [81], Chang et al. (2009: 10) [17], Chang et al. (2009: 1434) [91], Dong & Huang (2009: 103) [22], Kirejtshuk et al. (2010: 791) [82], Dong & Huang (2011: 1225) [79], Sohn et al. (2019: 3) [28], Yu et al. (2019: 381) [80].

Genus *Sinolithomerus* Dong & Huang, 2009

*Sinolithomerus* Dong & Huang, 2009: 103 [22]. Gender: masculine. Type species: *Sinolithomerus dolini* Dong & Huang, 2009: 104 [22]; by original designation. Jurassic of China (166.1–157.3 Ma). Monotypic.

Literature. Dong & Huang (2009: 103) [22], Dong & Huang (2011: 1225) [79], Yu et al. (2019: 383) [80].

- Protagrypninae *incertae sedis*

Genus *Paralithomerus* Chang, Zhang & Ren, 2008

*Paralithomerus* Chang, Zhang & Ren, 2008: 55 [81]. Gender: masculine. Type species: *Paralithomerus exquisitus* Chang, Zhang & Ren, 2008: 55 [81]; by original designation. Cretaceous of China (125.5–122.5 Ma). Two species.

Literature. Chang et al. (2008: 55) [81], Dong & Huang (2009: 103) [22], Kirejtshuk et al. (2010: 792) [82], Dong & Huang (2011: 1225) [79], Sohn et al. (2019: 3) [28], Yu et al. (2019: 382) [80].

Remark. This genus was originally described in Elateridae without any subfamilial assignment due to the presence of only one of two main diagnostic characters of Protagrypninae [81]. However, subsequent authors placed *Paralithomerus* into Protagrypninae [28,82].

#### 3.1.9. Elateridae *incertae sedis*

Genus *Adocetus* Scudder, 1900

*Adocetus* Scudder, 1900: 97 [92]. Gender: masculine. Type species: *Adocetus buprestoides* Scudder, 1900: 97 [92]; by monotypy. Eocene of the United States (55.8–50.3 Ma). Monotypic.

Literature. Scudder (1900: 97) [92], Handlirsch (1907: 747) [93], Carpenter (1992: 304) [38].

Remark. Both Scudder [92] and Carpenter [38] hypothesized the close relationship between *Adocetus* and *Scaptolenus* LeConte, 1853 (Elaterinae: Cebrionini) but the shape of prothorax in *Adocetus* is completely different.

Genus *Artinama* Lin, 1986

*Artinama* Lin, 1986: 72 [21]. Gender: feminine. Type species: *Artinama qinghuoensis* Lin, 1986: 73 [21]; by original designation. Jurassic of China (199.3–190.8 Ma). Monotypic.

Literature. Lin (1986: 72) [21], Dong & Huang (2011: 1225) [79], Dong et al. (2011: 482) [87], Ponomarenko et al. (2012: 477) [83], Yu et al. (2019: 410) [80].

Remark. It was originally placed in Acanthocnemidae, although with a question mark [21]. Dong et al. [87] placed it in Elateridae, again with a question mark. Ponomarenko et al. [83] hypothesized that *Artinama* belongs either to Praelateridae (currently synonymized with Cerophytidae by Yu et al. [94]) or Elateridae: Protagrypninae.

Genus *Bilineariselater* Chang & Ren, 2008

*Bilineariselater* Chang & Ren, 2008: 237 [24]. Gender: masculine. Type species: *Bilineariselater foveatus* Chang & Ren, 2008: 237 [24]; by original designation. Cretaceous of China (125.5–122.5 Ma). Monotypic.

Literature. Chang & Ren (2008: 237) [24], Kirejtshuk et al. (2010: 792) [82], Dong & Huang (2011: 1225) [79], Yu et al. (2019: 382) [80].

Remark. Chang & Ren [24] did not assign this genus to any subfamily due to the lack of diagnostic characters, although they hypothesized its relationship to either Agrypninae or Elaterinae. Kirejtshuk et al. [82] listed this genus under Protagrypninae but without any justification or clarification. Therefore, we here follow the original placement by Chang & Ren [24].

Genus *Cretoelaterium* Alekseev, 2008

*Cretoelaterium* Alekseev, 2008: 56 [95]. Gender: neuter. Type species: *Cretoelaterium kazanovense* Alekseev, 2008: 57 [95]; by original designation. Cretaceous of Russian Federation (129.4–125.0 Ma). Monotypic.

Literature. Alekseev (2008: 56) [95].

Remark. Alekseev [95] classified this genus in Elateridae without any subfamilial assignment.

Genus *Cryptocoelus* Dolin & Nel, 2002

*Cryptocoelus* Dolin & Nel, 2002: 342 [89]. Gender: masculine. Type species: *Cryptocoelus buffoni* Dolin & Nel, 2002: 342 [89]; by original designation (not *C. major* Dolin & Nel, 2002: 343 as erroneously stated by Korneev & Cate [46] and Chang et al. [56]). Cretaceous of China (125.5–122.5 Ma), Cretaceous of Russian Federation (125.0–113.0 Ma). Eight species.

*Cryptocoleous*: Chang et al., 2007: 1245 [56]; unavailable name, incorrect subsequent spelling not in prevailing usage [41].

*Crytocoelus*: Yu et al., 2019: 381 [80]; unavailable name, incorrect subsequent spelling not in prevailing usage [41].

Literature. Dolin & Nel (2002: 342) [89], Korneev & Cate (2005: 10) [46], Chang et al. (2007: 1245) [56], Chang & Ren (2008: 237) [24], Chang et al. (2009: 8) [17], Dong & Huang (2009: 102) [22], Chang et al. (2010: 873) [25], Kirejtshuk et al. (2010: 792) [82], Alekseev (2011: 424) [26], Dong & Huang (2011: 1225) [79], Yu et al. (2019: 381) [80].

Remark. This genus was originally classified in the agrypnine tribe Cryptocardiini [89], and its placement in Agrypninae was followed by Kirejtshuk et al. [82]. However, Chang et al. [56] revised the morphology of *Cryptocoelus* and classified it in Elateridae *incertae sedis*, which was followed by Alekseev [26].

Genus *Curtelater* Chang & Ren, 2008

*Curtelater* Chang & Ren, 2008: 238 [24]. Gender: masculine. Type species: *Curtelater wui* Chang & Ren, 2008: 239 [24]; by original designation. Cretaceous of China (125.5–122.5 Ma). Monotypic.

Literature. Chang & Ren (2008: 238) [24], Kirejtshuk et al. (2010: 792) [82], Dong & Huang (2011: 1225) [79], Yu et al. (2019: 382) [80].

Remark. Chang & Ren [24] did not assign this genus to any subfamily because of the lack of diagnostic characters, although they hypothesized its relationship to either Agrypninae or Elaterinae. Kirejtshuk et al. [82] listed this genus under Protagrypninae but without any justification or clarification. Therefore, we here follow the original placement by Chang & Ren [24].

Genus *Elateridium* Tillyard, 1918

*Elaterites* Tillyard, 1916: 41 [96]. Preoccupied by *Elaterites* Heer, 1847: 141 [97] [Coleoptera: Elateridae]. *Elateridium* Tillyard, 1918: 751 [98]. Replacement name for *Elaterites* Tillyard, 1916. Gender: neuter. Type species: *Elaterites wianamattensis* Tillyard, 1916: 41 [96]; by original designation. Triassic of Australia (247.2–208.5 Ma). Three species.

*Elaterium*: Ponomarenko, 2011: 421 [99]; unavailable name, incorrect subsequent spelling of *Elateridium* Tillyard, 1918, not in prevailing usage [41]. Preoccupied by *Elaterium* Westwood, 1854.

Literature. Tillyard (1916: 41) [96], Tillyard (1918: 751) [98], Dunstan (1923: 44) [100], Handlirsch (1938: 13) [101], Carpenter (1992: 304) [38], Jell (2004: 76) [102], Martins-Neto et al. (2006: 602) [103], Ponomarenko (2011: 421) [99].

Remark. This genus contains species described based on a single elytron each [96,100], and its systematic placement remains unclear.

Genus *Elaterites* Heer, 1847

*Elaterites* Heer, 1847: 141 [97]. Gender: masculine. Type species: *Elaterites lavateri* Heer, 1847: 141 [97]; by subsequent designation (Handlirsch 1907: 747 [93]). Paleocene of Argentina (66.0–56.0 Ma), Eocene of the United Kingdom (56.0–41.3 Ma), Eocene of Germany (47.8–41.3 Ma), Eocene of Czech Republic (37.2–33.9 Ma), Oligocene of Switzerland (28.4–23.0 Ma), Miocene of Germany (12.7–11.6 Ma). 12 species.

Literature. Heer (1847: 141) [97], Giebel (1856: 94) [85], Deichmüller (1881: 308) [104], Scudder (1885: 797) [105], Scudder (1886: 77) [106], Scudder (1891: 205) [34], Scudder (1900: 98) [92], Handlirsch (1906: 450) [35], Handlirsch (1907: 747) [93], Cockerell (1920: 456) [107], Dunstan (1923: 44) [100], Cockerell (1926: 320) [108], Haupt (1956: 48) [58], Martins-Neto et al. (2006: 602) [103].

Remark. Heer [97] erected this genus to accommodate the species which he was unable to place to any existing genus at that time. This genus is a waste basket for fossils with no clear characters (mostly represented by isolated elytra) and may contain representatives of many genera [36,97,107].

Genus *Elaterium* Westwood, 1854

*Elaterium* Westwood, 1854: 387/393 [109]. Gender: masculine. Type species: *Elaterium pronaeus* Westwood, 1854: 387/393 [109]; by subsequent monotypy in Handlirsch (1906: 553) [35], not by subsequent designation [36]. Triassic of Australia (228.0–208.5 Ma), Cretaceous of the United Kingdom (145.0–140.2 Ma). Two species.

Literature. Westwood (1854: 387/393) [109], Giebel (1856: 92) [85], Scudder (1885: 797) [105], Scudder (1886: 77) [106], Scudder (1891: 518) [34], Handlirsch (1906: 553) [35], Handlirsch (1907: 748) [93], Hyslop (1921: 644) [36], Dunstan (1923: 46) [100], Cockerell (1920: 456) [107], Handlirsch (1938: 14) [101], Jell (2004: 76) [102], Coram & Jepson (2012: 60) [110], Kirejtshuk (2020: 18) [111].

Remark. This genus has never been classified to any existing elaterid subfamily and its position remains unclear.

Genus *Gripecolous* Lin, 1986

*Gripecolous* Lin, 1986: 80 [21]. Gender: masculine. Type species: *Gripecolous enallus* Lin, 1986: 80 [21]; by original designation. Jurassic of China (170.3–168.3 Ma). Monotypic.

Literature. Lin (1986: 80) [21], Dong & Huang (2011: 1225) [79], Dong et al. (2011: 482) [87], Ponomarenko et al. (2012: 482) [83], Yu et al. (2019: 410) [80].

Remark. This genus was originally described in Silphidae [21]. Dong et al. [87] listed it in Protagrypninae, Ponomarenko et al. [83] hypothesized that *Gripecolous* belongs either to Praelateridae or Elateridae: Protagrypninae, and Yu et al. [80] listed it in Elateridae without further details.

Genus *Ludiophanes* Wickham, 1916

*Ludiophanes* Wickham, 1916: 522 [29]. Gender: masculine. Type species: *Ludiophanes haydeni* Wickham, 1916: 522 [29]; by original designation. Eocene of the United States (38.0–33.9 Ma). Monotypic.

Literature. Wickham (1916: 522) [29], Hyslop (1921: 654) [36], Carpenter (1992: 305) [38].

Remark. This genus has never been classified to any existing elaterid subfamily although Wickham [29] and Carpenter [38] hypothesized its affinities to *Ludius* Latreille, 1834 (currently a synonym of *Elater* Linnaeus, 1758) and *Megapenthes* Kiesenwetter, 1858, respectively, which both are currently classified in Elaterinae.

Genus *Mercata* Lin, 1986

*Mercata* Lin, 1986: 79 [21]. Gender: feminine. Type species: *Mercata festira* Lin, 1986: 79 [21]; by original designation. Jurassic of China (170.3–168.3 Ma). Monotypic.

Literature. Lin (1986: 79) [21], Chang et al. (2011: 33) [84], Chang et al. (2011: 701) [112], Dong & Huang (2011: 1225) [79], Dong et al. (2011: 486) [87], Ponomarenko et al. (2012: 480) [83], Oberprieler et al. (2016: 177) [113], Kundrata & Jäch (2017: 371) [114], Yu et al. (2019: 410) [80].

Remarks. This genus was originally classified in Silphidae [21] but later transferred to Cerophytidae by Chang et al. [84]. The latter authors provided no evidence for the placement of *Mercata* in Cerophytidae, and in other publication they wrote that it is questionable [112]. *Mercata* was not included in the most recent revision of the fossil Cerophytidae [94]. Ponomarenko et al. [83] classified *Mercata* in Elateridae: Protagrypninae without any tribal assignment, and Yu et al. [80] placed it in Elateridae without further details.

Genus *Ovivagina* Zhang, 1997

*Ovivagina* Zhang, 1997: 71 [88]. Gender: feminine. Type species: *Ovivagina longa* Zhang, 1997: 72 [88]; by original designation. Jurassic of China (201.3–190.8 Ma). Monotypic.

Literature. Zhang (1997: 71) [88], Dolin & Nel (2002: 345) [89], Chang et al. (2009: 8) [17], Dong & Huang (2009: 102) [22], Yan & Zhang (2010: 451) [115], Dong & Huang (2011: 1225) [79], Yan et al. (2013: 43) [116], Yu et al. (2019: 410) [80].

Remark. Zhang [88] described five species of this genus, but all of them except the type species, *O. longa*, were transferred to the genus *Artematopodites* Ponomarenko, 1990 [115], which has an uncertain position within Coleoptera [117,118,119]. The placement of *O. longa* in Elateridae is also dubious [17,79,115].

Genus *Protocardiophorus* Dolin, 1976

*Protocardiophorus* Dolin, 1976: 71 [20]. Gender: masculine. Type species: *Protocardiophorus ancestralis* Dolin, 1976: 73 [20]; by original designation. Jurassic of Kazakhstan (166.1–157.3 Ma). Two species.

Literature. Dolin (1976: 71) [20], Dolin (1980: 78) [13], Carpenter (1992: 305) [38], Korneev & Cate (2005: 10) [46], Douglas (2011: 19) [74], Douglas (2017: 4) [63].

Remark. This genus was originally placed in Cardiophorinae [13,20], however, Douglas [74] did not confirm this placement based on the morphological phylogenetic analysis.

Genus Pseudocardiophorites Dolin, 1976

*Pseudocardiophorites* Dolin, 1976: 73 [20]. Gender: masculine. Type species: *Pseudocardiophorites fragilis* Dolin, 1976: 73 [20]; by original designation. Jurassic of Kazakhstan (166.1–157.3 Ma). Five species.

Literature. Dolin (1976: 73) [20], Dolin (1980: 79) [13], Carpenter (1992: 305) [38], Korneev & Cate (2005: 10) [46], Douglas (2011: 19) [74], Douglas (2017: 4) [63].

Remark. This genus was originally placed in Cardiophorinae [13,20], however, Douglas [74] did not confirm this placement based on the morphological phylogenetic analysis.

Genus *Silicernius* Heyden, 1859

*Silicernius* Heyden, 1859: 6 [120]. Gender: masculine. Type species: *Silicernius spectabilis* Heyden, 1859: 6 [120]; by monotypy. Oligocene of Germany (28.4–23.0 Ma). Monotypic.

Literature. Heyden (1859: 6) [120], Scudder (1885: 797) [105], Scudder (1886: 77) [106], Scudder (1891: 580) [34], Handlirsch (1907: 747) [93], Hyslop (1921: 669) [36].

Remark. This genus has never been classified to any existing elaterid subfamily and its position remains unclear.

Genus *Sinoelaterium* Ping, 1928

*Sinoelaterium* Ping, 1928: 22 [23]. Gender: neuter. Type species: *Sinoelaterium melanocolor* Ping, 1928: 23 [23]; by original designation. Cretaceous of China (125.5–122.5 Ma). Monotypic.

Literature. Ping (1928: 22) [23], Handlirsch (1938: 167) [101], Carpenter (1992: 305) [38], Dolin & Nel (2002: 345) [89], Chang et al. (2009: 8) [17], Dong & Huang (2009: 102) [22], Dong & Huang (2011: 1225) [79], Yu et al. (2019: 409) [80].

Remark. Ping [23] described *Sinoelaterium* in Elateridae. Crowson [121] and Hörnschemeyer [122] hypothesized that it might belong to Artematopodidae. However, most authors placed *Sinoelaterium* into Elateridae, although with some reservations [38,80,101].

Genus *Tetraraphes* Iablokoff-Khnzorian, 1961

*Tetraraphes* Iablokoff-Khnzorian, 1961: 95 [32]. Gender: masculine. Type species: *Tetraraphes ebersini* Iablokoff-Khnzorian, 1961: 96 [32]; by original designation. Eocene Baltic amber (38.0–33.9 Ma). Monotypic.

Literature. Iablokoff-Khnzorian (1961: 95) [32], Larsson (1978: 153) [54], Zherikhin (1980: 60) [123], Spahr (1981: 49) [37], Carpenter (1992: 305) [38], Chang et al. (2010: 867) [25], Alekseev (2013: 7) [39], Alekseev (2017: 409) [55].

Remark. This genus was originally described in Elateridae without any subfamilial assignment, and this was followed by subsequent authors. Chang et al. [25] discussed the morphological characters of *Tetraraphes* and its similarity in the shape of metacoxal plates to Desmatini but also listed characters in which these taxa differ.

Genus *Turonelater* Alekseev, 2011

*Turonelater* Alekseev, 2011: 430 [26]. Gender: masculine. Type species: *Turonelater giganteus* Alekseev, 2011: 430 [26]; by original designation. Cretaceous of Kazakhstan (93.9–89.8 Ma). Monotypic.

Literature. Alekseev (2011: 430) [26].

Remark: This genus was classified as Elateridae *incertae sedis* by Alekseev [26].

### 3.2. Fossil Genera Excluded Here from Elateridae

Genus *Babuskaya* Martins-Neto & Gallego, 2009

*Babuskaya* Martins-Neto & Gallego, 2009: 368 [124]. Gender: feminine. Type species: *Babuskaya elaterata* Martins-Neto & Gallego, 2009: 368 [124]; by original designation. Triassic of Argentina (237.0–228.0 Ma). Monotypic.

Literature. Martins-Neto & Gallego (2009: 368) [124], Martins-Neto et al. (2011: 3) [125], Lara et al. (2012: 6) [126].

Remark. Martins-Neto & Gallego [124] placed this genus in Elateridae with a question mark. Since the elytron, based on which this genus was described, shows none of the diagnostic characters of Elateridae, we transfer *Babuskaya* Martins-Neto & Gallego, 2009 to Coleoptera *incertae sedis*.

Genus *Cardiosyne* Martins-Neto & Gallego in Martins-Neto et al., 2006

*Cardiosyne* Martins-Neto & Gallego in Martins-Neto et al., 2006: 602 [103]. Gender: feminine. Type species: *Cardiosyne obesa* Martins-Neto & Gallego in Martins-Neto et al., 2006: 602 [103]; by original designation. Triassic of Argentina (237.0–228.0 Ma). Two species.

Literature. Martins-Neto et al. (2006: 602) [103], Martins-Neto et al. (2011: 3) [125], Lara et al. (2012: 6) [126].

Remark. Martins-Neto et al. [103] classified this genus in Elateridae only tentatively and hypothesized that it might in fact belong to a yet undescribed beetle family. Since the elytron, based on which this genus was described, shows none of the diagnostic characters of Elateridae, we transfer *Cardiosyne* Martins-Neto & Gallego, 2006 to Coleoptera *incertae sedis*.

Genus *Fengningia* Hong, 1984

*Fengningia* Hong, 1984: 167 [127]. Gender: feminine. Type species: *Fengningia punctata* Hong, 1984: 167 [127]; by original designation. Cretaceous of China (125.0–113.0 Ma). Monotypic.

Literature. Hong (1984: 167) [127], Dong & Huang (2011: 1225) [79], Yu et al. (2019: 409) [80].

Remark. The habitus line-drawing of the holotype by Hong [127] shows none of the diagnostic characters of Elateridae, which was already discussed by Dong & Huang [79]. Therefore, we transfer *Fengningia* Hong, 1984 to Coleoptera *incertae sedis*.

Genus *Gemelina* Martins-Neto & Gallego in Martins-Neto et al., 2006

*Gemelina* Martins-Neto & Gallego in Martins-Neto et al., 2006: 602 [103]. Gender: feminine. Type species: *Gemelina triangularis* Martins-Neto & Gallego in Martins-Neto et al., 2006: 602 [103]; by original designation. Triassic of Argentina (237.0–228.0 Ma). Monotypic.

Literature. Martins-Neto et al. (2006: 602) [103], Martins-Neto et al. (2011: 3) [125], Lara et al. (2012: 6) [126].

Remark. Martins-Neto et al. [103] placed this genus in Elateridae with a question mark. Since the elytron, based on which this genus was described, shows none of the diagnostic characters of Elateridae, we transfer *Gemelina* Martins-Neto & Gallego, 2006 to Coleoptera *incertae sedis*.

## 4. Discussion

Elateridae are among the most common beetle families in the fossil record [16,54,128]. However, their real palaeodiversity remains understudied [16], most probably because of their rather uniform and generally problematic external morphology which often causes problems even in identification and classification of recent lineages [1,3,4,5,6,7,8,9,10,11]. Consequently, the systematics and classification of fossil elaterids has been in a constant state of flux. Many genera that were listed as belonging to Elateridae by various authors in the past, have been currently classified in different beetle families, or they bear no diagnostic characters which would allow researchers to place them properly, and so they stay as *incertae sedis*.

Handlirsch [35,93] listed in Elateridae many doubtful genera, usually described based on a single elytron, which can belong to any beetle family. This causes problems not only in Elateridae; for example, Nabozhenko [129] pointed out this issue and did not include Handlirsch’s taxa in his catalogue of fossil Tenebrionidae. Although the systematic position of many genera listed by Handlirsch [35,93] were not clear and some placements were questioned even by Handlirsch himself, Hyslop [36] listed many of them in his catalogue of the generic names in Elateridae, and added some more. The genera *Apistotes* Handlirsch, 1906, *Biadelater* Handlirsch, 1906, *Parabuprestium* Handlirsch, 1906, *Micrelaterium* Handlirsch, 1906 and *Paragrilium* Handlirsch, 1906 from the Cretaceous of the United Kingdom, *Anepismus* Handlirsch, 1906, *Mimelater* Handlirsch, 1906 and *Stenelytron* Handlirsch, 1906 from the Triassic of the United Kingdom, *Dysarestus* Handlirsch, 1906, *Glaphyropterites* Handlirsch, 1906 and *Megacentrus* Heer, 1852 from the Jurassic of Switzerland, *Enamma* Handlirsch, 1906 from the Jurassic of Germany, *Pseudoelateropsis* Handlirsch, 1906 (the replacement name for *Elateropsis* Roemer, 1876) from the Triassic of Germany, *Plastelater* Handlirsch, 1906 from the Triassic of the United Kingdom and Jurassic of Russian Federation, and *Keleusticus* Handlirsch, 1906 from the Jurassic of Germany, Mongolia and Russian Federation, are currently classified in Coleoptera *incertae sedis* [38]. *Anchylocheira* Giebel, 1856 was in fact a misspelling of *Ancylochira* Eschscholtz, 1829, which is currently in Buprestidae, and *Hydriphilites* Geinitz, 1884 was a misspelling of *Hydrophilites* Heer, 1865, which is classified in Coleoptera *incertae sedis* [38]. *Glaphyroptera* Heer, 1852 from the Jurassic of Switzerland was moved to its own family Glaphyropteridae [130] and later transferred to Buprestidae [44,131]. *Pseudothyrea* Handlirsch, 1906 from the Jurassic of Germany was also placed in Buprestidae [131,132]. *Glaphyropterodes* Handlirsch, 1906 from the Jurassic of Switzerland and Russian Federation was classified in Coleoptera *incertae sedis* [38,133] and currently it has been placed in the extinct elateriform family Lasiosynidae [118]. *Malmelater* Handlirsch, 1906 from the Jurassic of Germany, which was hypothesized by older authors to be the first fossil reliably placed in Elateridae [29,35], was transferred to Phoroschizidae (= Schizophoridae) [38,134]. The monotypic *Mecynocanthus* Hope, 1837 from the Holocene tropical fossil resin was synonymized with the extant genus *Agrypnus* Eschscholtz, 1829 [135].

Besides the problematic genera catalogued by Hyslop [36], there were a few other fossil genera classified in Elateridae which are currently considered not belonging to this family. Martynov [136] described genus *Tersus* from the Jurassic of Kazakhstan, in Elateridae. Although Crowson [121] suggested this genus might belong to Ptilodactylidae, it has been currently classified in the archostematan family Phoroschizidae (= Schizophoridae) [38,137,138]. The genus *Idiomerus* Dolin, 1980 from the Jurassic of Kazakhstan was originally described in Elateridae [13,38] but later synonymized with the genus *Necromera* Martynov, 1926 (Cerophytidae) by Chang et al. [84], which was followed by subsequent authors [80,94,113,114]. The monotypic *Elaterina* Gardiner, 1961 from the Jurassic of the United Kingdom was originally placed in Elateridae, but Whalley [14] questioned that placement because the holotype elytron of *E. liassica* Gardiner, 1961 shows none of the diagnostic characters of Elateridae. Carpenter [38] classified it in Coleoptera *incertae sedis*. The position of *Fengningia* Hong, 1984 (Cretaceous of China) [127] in Elateridae was questioned by Dong & Huang [79], and this genus is transferred in this study to Coleoptera *incertae sedis*. The genus *Macronotus* Hong & Wang, 1990 from the Cretaceous of China was originally placed in Buprestidae [139] but Dong & Huang [79] suggested it might belong to Elateridae based on the shape of elytra. However, for example Ding et al. [140] and Kirejtshuk & Ponomarenko [33] listed this genus again in Buprestidae. Three genera from the Triassic of Argentina, which were described based on the elytra only, i.e., *Babuskaya*, *Cardiosyne* and *Gemelina* [103,124], were originally classified in Elateridae only provisionally (with a question mark), and all of them are placed in Coleoptera *incertae sedis* in this study. Additionally, Dong et al. [87] listed in Elateridae *Mesagyrtes* Ponomarenko, 1977 from the Jurassic of the Russian Federation, although with a question mark. *Mesagyrtes* Ponomarenko, 1977 was preoccupied by *Mesagyrtes* Broun, 1895, and therefore, the former name was replaced by *Mesecanus* Newton, 1982. This genus is currently classified in the staphylinoid family Agyrtidae as already proposed in the original publication [141]. Another genus name proposed in Elateridae was *Pseudoelater* Heer, 1847 [97], but this is an unavailable name (*nomen nudum*) according to the Code [41], because no species was assigned to it.

The current composition of fossil Elateridae includes 74 extinct genera and three subgenera. Fifty-five genera are classified in eight subfamilies, and additional 19 ones have an uncertain position within Elateridae, and remain as *incertae sedis*. Almost half of genera are included in the only extinct subfamily, Protagrypninae, which consists of four tribes [13,18,19,26,28]. This subfamily is characterized by the unique prosternum and mesoventrite but is in need of revision; some species originally placed there by Dolin [13] were already moved to Cerophytidae, and some other need to be thoroughly re-examined [81,84]. Regarding the extant elaterid subfamilies, five most species-rich ones, which together form the bulk of the recent click-beetle diversity, are represented in fossil record by some extinct genera. These subfamilies are widely accepted and cosmopolitan Elaterinae, Dendrometrinae, Agrypninae, Cardiophorinae, and Negastriinae. However, the numbers of fossil genera assigned to extant subfamilies are not very high, and in some cases even their placement is debatable. This is not surprising, because the morphological limits of many elaterid subfamilies and tribes are uncertain and there is no reliable identification key to higher taxa which would be valid worldwide. Often, there are no clear synapomorphies available even for the apparently monophyletic groups defined by the molecular-based phylogenetic methods [1,9,10]. Moreover, many higher taxa are defined by the combination of characters including some which are usually not available for the fossils, such as the larval morphology, wing venation, etc., [4,9,78]. It is especially not easy to assign the compression fossils into a proper subfamily, and this is particularly true for the large subfamilies Elaterinae and Dendrometrinae, whose adults can be usually (but not always) recognized by the shape of fronto-clypeal region [1,5]. Maybe this is the reason why both above-mentioned subfamilies include mainly taxa described from the amber inclusions rather than the compressions [32]. Elaterinae includes five fossil genera and one subgenus, and Dendrometrinae includes two genera and two subgenera. However, the positions of some of them need to be re-examined. For example, the genus *Cryptagriotes* Wickham, 1916, described based on the compression fossil from the Eocene of the United States, was hypothesized to be similar either to *Cryptohypnus* Eschscholtz, 1830 (syn. of *Hypnoidus* Dillwyn, 1829 [69]) (Dendrometrinae: Hypnoidini) or *Agriotes* Eschscholtz, 1830 (Elaterinae: Agriotini) [29,38]. Another large subfamily, Agrypninae, is represented by seven genera classified in three tribes, one of which is a monogeneric fossil Cryptocardiini. Although Dolin & Nel [89] placed another genus, *Cryptocoelus*, into this tribe, Chang et al. [56] later removed it from there, and also from Agrypninae, based on the different morphology of scutellum and prosternum. Three fossil genera usually classified in Cardiophorinae [13,20,61] were critically revised by Douglas [63,74]; two of them were removed from the subfamily, and the placement of the third one remains dubious. The closely related subfamily Negastriinae includes only two described fossil genera [13,20]. The smaller elaterid subfamilies, which include fossil genera, are represented by Omalisinae and Pityobiinae. The first one was placed into Elateridae only recently based on the molecular phylogeny [8], which explains why its only fossil genus, *Jantarokrama*, was earlier listed in Omalisidae [55,77].

The exact age of origin for Elateridae remains unclear, but there are many indications that they were already present in the Triassic Period [12,14,15]. Undescribed elaterids are reported from the Madygen Formation in southwest Kyrgyzstan, which dates back to Ladinian of Middle Triassic (242.0–228.0 Ma) [15,128]. The fossil record of Elateridae from the Triassic is somewhat problematic, because usually only body fragments, mostly elytra, are known for vast majority of species, and in the past, many doubtful specimens were designated as elaterids [35,93]. Genera *Babuskaya*, *Cardiosyne*, *Gemelina* (all from the Upper Triassic of Argentina, Carmian, ~230 Ma), *Mimelater*, *Plastelater*, *Stenelytron* (all from the Upper Triassic of the United Kingdom, Rhaetian, ~205 Ma), and *Pseudoelateropsis* (Triassic of Germany, Rhaetian, ~205 Ma) cannot be classified in Elateridae with certainty, and so they are currently considered Coleoptera *incertae sedis* [38]. The Triassic record of Elateridae which is based on elytra only, needs further investigation and should be interpreted with caution. This includes *Elaterium bipunctatum* Dunstan, 1923 and all three species of *Elateridium* from the Triassic of Australia (Norian, ~220 Ma and Anisian, ~245 Ma), and *Elaterophanes acutus* Cockerell, 1915 from the Triassic of the United Kingdom (Rhaetian, ~205 Ma) [86,96,100]. The only named representative of the Triassic Elateridae, for which we have habitus information and not only a single elytron available, is *Elaterophanes vetustus* (Brodie, 1845) from the Rhaetian Lilstock Formation in England (208.5–201.3 Ma) [12,35,85]. While Triassic *Elaterium* and *Elateridium* cannot be reliably placed in any of the elaterid subfamilies, *Elaterophanes* has been classified in Protagrypninae [14]. This fossil subfamily became increasingly abundant and diverse later in the Middle Jurassic, as documented by the presence of almost 30 genera classified in the tribes Desmatini, Hypnomorphini, and Protagrypnini [13,14,17,19,21,22,79,91]. Some other lineages of Elateridae also diversified during the Jurassic Period, such as Agrypninae (Agrypnini, Cryptocardiini), Dendrometrinae (Dimini), and Negastriinae [13,20]. The diversification of elaterids continued through the Cretaceous Period [23,24,25,26,27,28,89,95], and many recent genera were established by the early Cenozoic [16,29,33,142]. It is remarkable that no genera of Elaterinae and Dendrometrinae, which are recently the most genus-rich subfamilies, have not been reported from the Mesozoic. The only exception is *Alaodima*, which is assigned to Dimini, but there is an ongoing debate if this group belongs to Dendrometrinae or forms a separate subfamily [13,72]. The missing record of Elaterinae and Dendrometrinae genera in the early evolution of Elateridae is most probably artificial, because they belong to the basal-most splits based on the results of recent molecular phylogenetic analyses [9,10,11]. This situation may be partly caused by the hard-to-observe morphological characters defining the subfamilies and their tribes [1,4,5,78], which are usually not visible even if the fossils are well-preserved. In badly preserved click-beetle fossils, it is often almost impossible to find any important diagnostic character which would allow researchers to classify it correctly to the higher taxonomic rank. Another problem might be the misidentifications of some described taxa and misinterpretations of their systematic placements, and also the general underexamination of the fossil record, especially from the Mesozoic amber deposits [16,27,128]. Genera which are currently considered *incertae sedis* in Elateridae should be carefully investigated in order to improve our knowledge on the palaeodiversity of the internal lineages in Elateridae, including Elaterinae and Dendrometrinae.

## 5. Conclusions

The systematics and classification of fossil Elateridae is in a state of flux. Recent increase in publications focusing on the extinct click-beetles brought many new discoveries [25,26,27,28,77,79], which sounds promising for future research in the group. However, much more attempt should be made to better explore the real palaeodiversity of Elateridae. First of all, the revisions of taxa on all taxonomic levels should be made, including the critical re-examination of the only fossil subfamily Protagrypninae, which currently includes by far the highest generic diversity in fossil click-beetles [13,81,84]. These revisions should also include the *incertae sedis* taxa, which include mainly lineages from the early evolution of Elateridae in the Mesozoic Era. Additionally, the so-far unnamed click-beetle fossils reported from various deposits [15,16,55,128] should be investigated in order to help us better understand the real diversity and estimate the origin of main click-beetle lineages. For example, the amber deposits (especially Baltic, Dominican, Lebanese, and Burmese) are highly understudied and may be of a great importance because of the much better preservation of specimens than in the case of compression fossils [16,27,39,55,128]. Recent revision of the fossil representatives of the morphologically similar family Cerophytidae revealed much greater diversity than previously believed [94], and this can be expected also in Elateridae. Our study of the fossil genus-group name concepts in Elateridae should serve future scholars as a robust framework for further research on the palaeodiversity and evolution of the click-beetles.

## Figures and Tables

**Table 1:** Overview of the fossil genera and subgenera in Elateridae.

Subfamily Tribe	Genus	Period/Epoch, Location	Age (Ma)	Nr. of Species
**Agrypninae**				
Agrypnini	*Ageratus* Dolin, 1980	Jurassic of Kazakhstan	166.1–157.3	2
	*Compsoderus* Dolin, 1980	Jurassic of Kazakhstan	166.1–157.3	1
	*Litholacon* Dolin, 1980	Jurassic of Kazakhstan	166.1–157.3	7
	*Macropunctum* Tröster, 1991	Eocene of Germany and United Kingdom	48.6–33.9	13
	*Plagioraphes* Iablokoff-Khnzorian, 1961	Eocene Baltic amber	38.0–33.9	1
Cryptocardiini	*Cryptocardius* Dolin, 1980	Jurassic of Kazakhstan	166.1–157.3	1
Pyrophorini	*Eopyrophorus* Haupt, 1950	Eocene of Germany	47.8–41.3	1
**Cardiophorinae**				
	*Mionelater* Becker, 1963	Miocene of Mexico	23.0–16.0	1
**Dendrometrinae**				
Dendrometrini	*Athousiomorphus* Iablokoff-Khnzorian, 1961 *	Eocene Baltic amber	38.0–33.9	1
	*Paralimonius* Iablokoff-Khnzorian, 1961 **	Eocene Baltic amber	38.0–33.9	1
Dimini	*Alaodima* Dolin, 1980	Jurassic of Kazakhstan	166.1–157.3	1
Hypnoidini	*Cryptagriotes* Wickham, 1916	Eocene of the United States	37.2 –33.9	1
**Elaterinae**				
Ampedini	*Octamenogonoides* Iablokoff-Khnzorian, 1961 ***	Eocene Baltic amber	38.0–33.9	1
Elaterini	*Diaraphes* Iablokoff-Khnzorian, 1961	Eocene Baltic amber	38.0–33.9	1
	*Elatron* Iablokoff-Khnzorian, 1961	Eocene Baltic amber	38.0–33.9	1
	*Holopleurus* Iablokoff-Khnzorian, 1961	Eocene Baltic amber	38.0–33.9	1
	*Orthoraphes* Iablokoff-Khnzorian, 1961	Eocene Baltic amber	38.0–33.9	1
*incertae sedis*	*Crioraphes* Iablokoff-Khnzorian, 1961	Eocene Baltic amber	38.0–33.9	1
**Negastriinae**				
	*Ganestrius* Dolin, 1976	Jurassic of Kazakhstan	166.1–157.3	2
	*Protoquasimus* Dolin, 1976	Jurassic of Kazakhstan	166.1–157.3	1
Omalisinae				
	*Jantarokrama* Kirejtshuk & Kovalev, 2015	Eocene Baltic amber	38.0–33.9	1
Pityobiinae				
	*Cretopityobius* Otto, 2019	Cretaceous of Myanmar	99.6–93.5	1
**Protagrypninae**				
Desmatini	*Anoixis* Chang, Kirejtshuk & Ren, 2010	Cretaceous of China	125.5–122.5	1
	*Apoclion* Chang, Kirejtshuk & Ren, 2010	Cretaceous of China	125.5–122.5	3
	*Desmatinus* Chang, Kirejtshuk & Ren, 2010	Cretaceous of China	125.5–122.5	1
	*Desmatus* Dolin, 1975	Jurassic of Kazakhstan	166.1–157.3	4
	*Paradesmatus* Chang, Kirejtshuk & Ren in Chang et al., 2009	Jurassic and Cretaceous of China	166.1–122.5	3
	*Plesiorhaphes* Dolin, 1980	Jurassic of Kazakhstan	166.1–157.3	1
Hypnomorphini	*Abrotus* Dolin, 1980	Jurassic of Kazakhstan	166.1–157.3	2
	*Adiagnostus* Dolin, 1980	Jurassic of Kazakhstan	166.1–157.3	3
	*Codemus* Dolin, 1980	Jurassic of Kazakhstan	166.1–157.3	10
	*Elaterophanes* Handlirsch, 1906	Triassic and Jurassic of the United Kingdom	208.5–189.6	3
	*Graciolacon* Dolin, 1980	Jurassic of Kazakhstan	166.1–157.3	1
	*Hypnomorphoides* Dolin, 1980	Jurassic of Kazakhstan	166.1–157.3	4
	*Hypnomorphus* Dolin, 1975	Jurassic of Kazakhstan	166.1–157.3	14
	*Idiomorphus* Dolin, 1980	Jurassic of Kazakhstan	166.1–157.3	2
	*Lapidiconides* Dolin, 1980	Jurassic of Kazakhstan	166.1–157.3	3
	*Lapidostenus* Dolin, 1980	Jurassic of Kazakhstan	166.1–157.3	5
	*Lithoptychus* Dolin, 1980	Jurassic of Kazakhstan	166.1–157.3	4
	*Lithosomus* Dolin, 1980	Jurassic of Kazakhstan	166.1–157.3	2
	*Necrocoelus* Dolin, 1980	Jurassic of Kazakhstan	166.1–157.3	1
	*Negastrioides* Dolin, 1980	Jurassic of Kazakhstan	166.1–157.3	4
	*Parahypnomorphus* Dolin, 1980	Jurassic of Kazakhstan	166.1–157.3	3
	*Platyelater* Dolin, 1980	Jurassic of Kazakhstan	166.1–157.3	4
Pollostelaterini	*Pollostelater* Alekseev, 2011	Cretaceous of Russian Federation	125.0–113.0	1
Protagrypnini	*Acheonus* Dolin, 1980	Jurassic of Kazakhstan	166.1–157.3	3
	*Archaeolus* Lin, 1986	Jurassic of China	170.3–168.3	1
	*Clavelater* Dong & Huang, 2011	Jurassic of China	166.1–157.3	1
	*Koreagrypnus* Sohn & Nam in Sohn et al., 2019	Cretaceous of South Korea	113.0–100.5	1
	*Lithocoelus* Dolin, 1975	Jurassic of Kazakhstan	166.1–157.3	2
	*Lithomerus* Dolin, 1980	Jurassic of Australia and Kazakhstan, Cretaceous of China	182.7–122.5	6
	*Megalithomerus* Sohn & Nam in Sohn et al., 2019	Cretaceous of South Korea	113.0–100.5	1
	*Micragrypnites* Dolin, 1973	Jurassic of Kyrgyzstan	201.3–190.8	1
	*Paragrypnites* Dolin, 1980	Jurassic of Kazakhstan	166.1–157.3	1
	*Paraprotagrypnus* Chang, Zhao & Ren, 2009	Jurassic of China	166.1–157.3	1
	*Protagrypnus* Dolin, 1973	Jurassic of Kyrgyzstan and China	201.3–157.3	2
	*Sinolithomerus* Dong & Huang, 2009	Jurassic of China	166.1–157.3	1
*incertae sedis*	*Paralithomerus* Chang, Zhang & Ren, 2008	Cretaceous of China	125.5–122.5	2
**Elateridae *incertae sedis***				
	*Adocetus* Scudder, 1900	Eocene of the United States	55.8–50.3	1
	*Artinama* Lin, 1986	Jurassic of China	199.3–190.8	1
	*Bilineariselater* Chang & Ren, 2008	Cretaceous of China	125.5–122.5	1
	*Cretoelaterium* Alekseev, 2008	Cretaceous of Russian Federation	129.4–125.0	1
	*Cryptocoelus* Dolin & Nel, 2002	Cretaceous of China and Russian Federation	125.5–113.0	8
	*Curtelater* Chang & Ren, 2008	Cretaceous of China	125.5–122.5	1
	*Elateridium* Tillyard, 1918	Triassic of Australia	247.2–208.5	3
	*Elaterites* Heer, 1847	Paleocene of Argentina, Eocene of the United Kingdom, Germany and Czech Republic, Oligocene of Switzerland, Miocene of Germany	66.0–11.6	12
	*Elaterium* Westwood, 1854	Triassic of Australia, Cretaceous of the United Kingdom	228.0–140.2	2
	*Gripecolous* Lin, 1986	Jurassic of China	170.3–168.3	1
	*Ludiophanes* Wickham, 1916	Eocene of the United States	38.0–33.9	1
	*Mercata* Lin, 1986	Jurassic of China	170.3–168.3	1
	*Ovivagina* Zhang, 1997	Jurassic of China	201.3–190.8	1
	*Protocardiophorus* Dolin, 1976	Jurassic of Kazakhstan	166.1–157.3	2
	*Pseudocardiophorites* Dolin, 1976	Jurassic of Kazakhstan	166.1–157.3	5
	*Silicernius* Heyden, 1859	Oligocene of Germany	28.4–23.0	1
	*Sinoelaterium* Ping, 1928	Cretaceous of China	125.5–122.5	1
	*Tetraraphes* Iablokoff-Khnzorian, 1961	Eocene Baltic amber	38.0–33.9	1
	*Turonelater* Alekseev, 2011	Cretaceous of Kazakhstan	93.9–89.8	1

* Subgenus of *Athous* Eschscholtz, 1829, ** Subgenus of *Limonius* Eschscholtz, 1829, *** Subgenus of *Ampedus* Dejean, 1833.
